# Up-regulation of ribosome biogenesis by *MIR196A2* genetic variation promotes endometriosis development and progression

**DOI:** 10.18632/oncotarget.11536

**Published:** 2016-09-15

**Authors:** Cherry Yin-Yi Chang, Ming-Tsung Lai, Yi Chen, Ching-Wen Yang, Hui-Wen Chang, Cheng-Chan Lu, Chih-Mei Chen, Carmen Chan, Ching Chung, Chun-Cheng Tseng, Tritium Hwang, Jim Jinn-Chyuan Sheu, Fuu-Jen Tsai

**Affiliations:** ^1^ Department of Obstetrics and Gynecology, China Medical University Hospital, Taichung, Taiwan; ^2^ Institute of Environmental Health, China Medical University, Taichung, Taiwan; ^3^ Department of Pathology, Taichung Hospital, Ministry of Health and Welfare, Taichung, Taiwan; ^4^ Human Genetic Center, China Medical University Hospital, Taichung, Taiwan; ^5^ The Institute of Basic Medical Sciences, National Cheng Kung University, Tainan, Taiwan; ^6^ Institute of Biomedical Sciences, National Sun Yat-sen University, Kaohsiung, Taiwan; ^7^ School of Medicine, China Medical University, Taichung, Taiwan; ^8^ School of Chinese Medicine, China Medical University, Taichung, Taiwan; ^9^ Department of Health and Nutrition Biotechnology, Asia University, Taichung, Taiwan; ^10^ School of Post-Baccalaureate Chinese Medicine, China Medical University, Taichung, Taiwan

**Keywords:** ribosome biogenesis, MIR196A2, polymorphism, endometriosis, ovarian cancer

## Abstract

Aberrant miRNA expression has been reported in endometriosis and miRNA gene polymorphisms have been linked to cancer. Because certain ovarian cancers arise from endometriosis, we genotyped seven cancer-related miRNA single nucleotide polymorphisms (MiRSNPs) to investigate their possible roles in endometriosis. Genetic variants in *MIR196A2* (rs11614913) and *MIR100* (rs1834306) were found to be associated with endometriosis development and related clinical phenotypes, such as infertility and pain. Downstream analysis of the *MIR196A2* risk allele revealed upregulation of rRNA editing and protein synthesis genes, suggesting hyper-activation of ribosome biogenesis as a driving force for endometriosis progression. Clinical studies confirmed higher levels of small nucleolar RNAs and ribosomal proteins in atypical endometriosis lesions, and this was more pronounced in the associated ovarian clear cell carcinomas. Treating ovarian clear cells with CX5461, an RNA polymerase I inhibitor, suppressed cell growth and mobility followed by cell cycle arrest at G2/M stage and apoptosis. Our study thus uncovered a novel tumorigenesis pathway triggered by the cancer-related *MIR196A2* risk allele during endometriosis development and progression. We suggest that anti-RNA polymerase I therapy may be efficacious for treating endometriosis and associated malignancies.

## INTRODUCTION

Endometriosis is a benign, yet debilitating, gynecological disease associated with chronic pelvic pain, dysmenorrhea and infertility. Affecting about 10% of reproductive-age females, endometriosis causes abnormal growth of endometrium-like tissues outside the uterine cavity [1]. These benign peritoneal surface growths, which can invade ectopically, mimic the progression of metastasis in malignant cancer, which is accompanied by angiogenesis and cell migration [2, 3]. Histopathological observations and genetic analyses have shown that both endometrioid and clear cell ovarian carcinomas arise from endometriosis [4, 5]. Although several hypotheses have been proposed regarding the etiology of endometriosis, the exact pathogenesis of the disease remains unclear. Multiple factors may be involved in an individual's susceptibility to endometriosis, including hormone aberrations, abnormal immune responses, environmental factors and individual anatomy, as well as genetic or epigenetic predisposition [1, 3, 6-9].

MicroRNAs (miRNAs) are small non-coding single-stranded RNAs that post-transcriptionally regulate a wide range of biological processes, including cellular differentiation, proliferation and apoptosis. Targeting mRNA transcripts by miRNAs accelerates its transcript degradation or represses the translation, depending on the degree of complementarity [10, 11]. Single-nucleotide polymorphisms (SNPs) in miRNAs or their binding targets have been associated with aberrant miRNA expression and carcinogenesis [12–14]. Microarray and functional studies revealed that miRNA levels are related to benign conditions, malignant diseases and fertility disorders of the female reproductive tract alike [15–18]. While no link has yet been established between miRNA gene polymorphisms and endometriosis, such polymorphisms might be crucial epigenetic factors influencing endometriosis susceptibility [8, 9, 19]. In this study, we identified an endometriosis-related SNP in *MIR196A2* and demonstrated its novel function in regulating the expression of several small nucleolar RNAs (snoRNAs) and ribosomal proteins (RPs).

Small nucleolar RNAs (snoRNAs) are non-coding RNAs with longer mature sequences (60–300 nt) than miRNAs. They can be divided into two major classes with distinct signature sequences, box C/D or box H/ACA, functioning as guiding components for small ribonucleoprotein particles, catalyzing rRNA 2′-O-methylation and pseudouridylation, respectively, through complementary recognition sequences [20, 21]. In the eukaryotic cell nucleolus, ribosomal RNA is post-transcriptionally edited by snoRNAs and subsequently cleaved to yield 18S, 5.8S and 28S rRNAs. These fragments are assembled into mature large and small RPs, preceding translocation to the cytoplasm [22]. Both snoRNAs and RPs are key regulators in ribosome biogenesis, which is especially crucial for cell cycle progression [23, 24]. Recent studies suggested that upregulation of snoRNAs and RPs controls human tumor development [25–27]. Perturbation of ribosome assembly by RNA polymerase I inhibition or snoRNA/RP silencing can arrest cell proliferation and induce apoptosis, and has been suggested as a novel strategy against malignant diseases [28, 29]. Our study describes novel roles for certain small non-coding RNAs and RPs in promoting endometriosis development and associated malignancy.

## RESULTS

### Risk association analysis of cancer-related SNPs in miRNA genes (MiRSNPs)

We selected seven non-redundant SNPs within miRNA regions, also known as MiRSNPs, with minor allele frequencies over 4% in the Han Chinese in Beijing (HCB) population (HapMap database: www.hapmap.org). These MiRSNPs function as risk factors for various cancer types in case-control studies (Supplementary Table S1). They are located within either pre- or mature miRNAs and could thus interfere with stability and folding. Our data indicate that genetic variations at rs1834306 in *MIR100* (*p*=3.5×10^−3^, OR: 1.64; 95% CI: 1.24–2.17) and rs11614913 in *MIR196A2* (*p*=3.5×10^−3^, OR: 1.65; 95% CI: 1.24–2.19) are associated with endometriosis risk (Table 1). The rs11614913 C allele appears to dominantly affect endometriosis susceptibility; patients with CC or CT genotypes are at increased risk for endometriosis (*p*=7×10^−4^, OR: 2.45; 95% CI: 1.54–3.51). The rs1834306 A allele recessively affects endometriosis susceptibility (*p*=9.1×10^−3^, OR: 2.17; 95% CI: 1.35–3.51) (Table 2). Although the rs7372209 T allele in *MIR26A1* was also associated with increased endometriosis risk (Table 1), and the reference C allele was protective against endometriosis development (Table 2), these differences were not significant after Bonferroni correction.

**Table 1:** Allele distributions of cancer-related MiRSNPs in Taiwanese patients with endometriosis and controls

miRNA	SNP	MAF^a^		OR^b^	95% CI^c^	Nominal P-value^d^,^f^	Corrected P-value^e^,^f^
		Patients (n=218)	Control (n=202)				
miR-100	rs1834306	46.76%	34.83%	1.64	1.24-2.17	0.0005***	0.0035**
miR-146a	rs2910164	39.79%	34.83%	0.80	0.47-1.38	0.49	1.00
miR-196a2	rs11614913	55.21%	42.79%	1.65	1.24-2.19	0.0005***	0.0035**
miR-26a1	rs7372209	36.27%	28.71%	1.41	1.05-1.91	0.0232*	0.1624
miR-27a	rs895819	24.06%	28.39%	0.86	0.62-1.19	0.3594	1.00
miR-423	rs6505162	21.24%	19.55%	1.11	0.78-1.57	0.5541	1.00
miR-499	rs3746444	42.66%	37.16%	1.26	0.90-1.75	0.1761	1.00

a MAF, minor allele frequency.

b OR, odds ratio of minor alleles with reference to major alleles.

c 95% CI, 95% confidence interval.

d *P*-values were calculated by chi-square tests.

e Bonferroni method was applied for multiple test correction.

f Statistical significance

* *P* < 0.05

** *P* < 0.01

*** *P* < 0.001).

**Table 2:** Genotype distributions of cancer-related MiRSNPs in Taiwanese patients with endometriosis and controls

SNP (miRNA)	Genotype	No.(%) of patients	No.(%) of control	P-value^a^,^c^	Corrected P-value^b^,^c^	OR (95% CI)^d^
rs1834306	AA	63 (29.2)	32 (15.9)	0.0039**	0.0273*	2.38 (1.41- 4.01)
(miR-100)	AG	76 (35.2)	76 (37.8)			1.21 (0.78-1.87)
	GG	77 (35.6)	93 (46.3)			1.00
	AA+AG	139(64.4)	108(53.7)	0.0275*	0.1918	1.55 (1.05-2.30)
	GG	77 (35.6)	93 (46.3)			1.00
	AA	63 (29.2)	32 (15.9)	0.0013**	0.0091**	2.17 (1.35-3.51)
	GG+AG	153 (70.8)	169 (84.1)			1.00
rs2910164	GG	38 (19.9)	32 (15.9)	0.4135	1.00	1.43 (0.82-2.51)
(miR-146a)	CG	76 (39.8)	76 (37.8)			1.21 (0.78-1.87)
	CC	77 (40.3)	93 (46.3)			1.00
	GG+CG	114 (59.7)	108 (53.7)	0.2344	1.00	1.27 (0.85-1.90)
	CC	77 (40.3)	93 (46.3)			1.00
rs11614913	CC	55 (28.6)	42 (20.9)	0.0006***	0.0042**	2.59 (1.47-4.58)
(miR-196a2)	CT	102 (53.2)	88 (43.8)			2.35 (1.43-3.86)
	TT	35 (18.2)	71 (35.3)			1.00
	CC+CT	157 (81.8)	130 (64.7)	0.0001***	0.0007***	2.45 (1.54-3.51)
	TT	35 (18.2)	71 (35.3)			1.00
rs7372209	TT	32 (16.6)	17 (8.4)	0.0419*	0.2938	2.28 (1.19-4.39)
(miR-26a-1)	CT	76 (39.4)	82 (40.6)			1.12 (0.73-1.72)
	CC	85 (44.0)	103 (51.0)			1.00
	TT+CT	108 (56.0)	99 (49.0)	0.0139*	0.0971	1.32 (0.89-1.46)
	CC	85 (44.0)	103 (51.0)			1.00
	TT	32 (16.6)	17 (8.4)	0.0139*	0.0971	2.16 (1.16-4.04)
	CC+CT	161 (83.4)	185 (91.6)			1.00
rs895819	CC	15 (8.0)	16 (8.0)	0.1962	1.00	0.85 (0.40-1.81)
(miR-27a)	CT	60 (32.1)	81 (40.7)			0.67 (0.44-1.04)
	TT	112 (59.9)	102 (51.3)			1.00
	CC+CT	75 (40.1)	97 (48.7)	0.0880	0.6158	0.70 (0.47-1.05)
	TT	112 (59.9)	102 (51.3)			1.00
rs6505162	AA	12 (6.2)	9 (4.5)	0.7345	1.00	1.43 (0.58-3.51)
(miR-423)	AC	58 (30.1)	61 (30.2)			1.02 (0.66-1.58)
	CC	123 (63.7)	132 (65.3)			1.00
	AA+AC	70 (36.3)	70 (34.7)	0.4352	1.00	1.07 (0.71-1.62)
	CC	123 (63.7)	132 (65.3)			1.00
rs3746444	CC	31 (21.6)	26 (17.6)	0.4430	1.00	1.47 (0.78-2.77)
(miR-499)	CT	60 (42.0)	58 (39.2)			1.27 (0.76-2.13)
	TT	52 (36.4)	64 (43.2)			1.00
	CC+CT	91 (63.6)	84 (56.8)	0.3771	1.00	1.33 (0.83-2.14)
	TT	52 (36.4)	64 (43.2)			1.00

a Genotype associations with endometriosis were determined by chi-square tests.

b Bonferroni method was applied for multiple test correction.

c Statistical significance

d OR, odds ratio of minor alleles with reference to major alleles; 95% CI, 95% confidence interval.

* *P* < 0.05

** *P* < 0.01

*** *P* < 0.001).

### Association of MiRSNPs with clinical phenotypes

**Figure 1:**
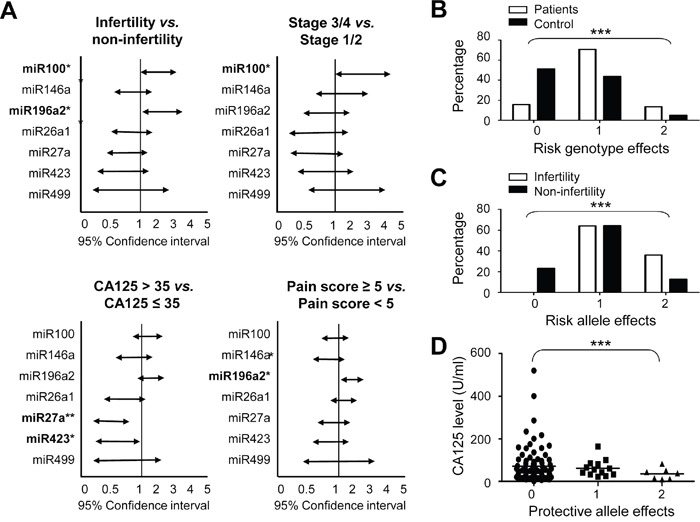
Risk analysis of cancer-related MiRSNPs for endometriosis and related clinical symptoms Allele distributions of the defined MiRSNPs in patients were analyzed by chi-square tests and represented as 95% confidence intervals according to the indicated endometriosis-associated clinical symptoms **A.** Combined genotype analysis of rs11614913 (CC or CT genotype in *MIR196A2*) and rs1834306 (AA genotype in *MIR100*) was performed for endometriosis risk prediction **B.** Combined allelic type analysis of rs11614913 (C allelie in *MIR196A2*) and rs1834306 (A allele in *MIR100*) was performed to predict endometriosis-associated infertility **C.** CA125 levels in patients with different protective allele effects were determined by combining rs895819 (C allele in *MIR27A*) and rs6505162 (A allele in *MIR423*) allelic types **D.** Combination effects were labeled as 0: objectives with no risk or protective genotype/allelic type from either MiRSNP; 1: objectives with one risk or protective genotype/allelic type from either MiRSNP; 2: objectives with risk or protective genotype/allelic types from both MiRSNPs. *P<0.05; **P<0.01; ***P<0.001.

Using patient records, we discovered a number of MiRSNPs linked to the development of endometriosis-associated phenotypes, including infertility, clinical stage, CA125 levels and pain scores (Figure 1A). The rs1834306 A allele in *MIR100*, which determines progression time in colon cancer [30], appeared linked to both infertility (*p*=0.040) and advanced endometriosis stage (*p*=0.041) (Figure 1A). The rs11614913 C allele in *MIR196A2* was involved in infertility (*p*=0.016) and increased pain severity (*p*=0.012), whereas SNP rs7372209 in *MIR26A1* was not associated with any clinical symptoms. The rs895819 in *MIR27A* and rs6505162 in *MIR423*, suggested to be protective alleles against endometriosis (Table 2), were linked with reduced CA125 levels (*p*=0.0058 and 0.039, respectively; Figure 1A). Our data confirm the association of *MIR100* and *MIR196A2* genetic variants with endometriosis risk and cancer development [30–33].

We used a disease-associated genotype analysis to assess possible cumulative effects for the two pro-endometriosis functional SNPs, rs11614913 (CC or CT) of *MIR196A2* and rs1834306 (AA) of *MIR100*. Data indicated that patients and controls had distinct cumulative risk scores (*p*<10^−5^; Figure 1B). Compared to low-risk patients with zero unfavorable genotypes, medium-risk patients with one unfavorable genotype had an OR of 5.31 (95% CI: 3.26–8.66), and high-risk patients with two unfavorable genotypes had an OR of 8.84 (95% CI: 4.06–19.2; Supplementary Table S2). Similarly, a combination of the risk alleles at rs11614913 (C) in *MIR196A2* and rs1834306 (G) in *MIR100* predicted endometriosis-related infertility (*p*<0.001; Figure 1C). No patient with zero risk alleles developed infertility in our study group. By contrast, a combination of the minor allele frequencies in *MIR27A* and *MIR423* divided patients into three groups with differentt CA125 levels (*p*<0.001; Figure 1D).

### Variations at rs11614913 in *MIR196A2* lead to rRNA editing/modification and protein synthesis malfunction in endometrial cells

**Figure 2:**
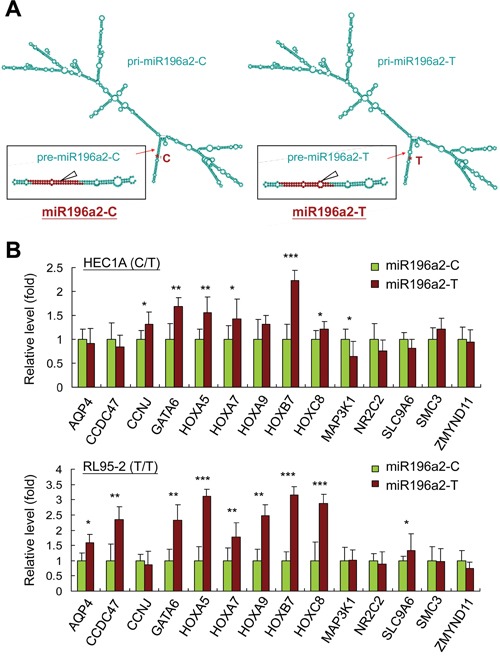
MIR196A2 genetic variant affects on RNA structures and downstream target gene expression The predicted pri-miRNA and pre-miRNA structures of miR196a2 with genetic variations at rs11614913 were analyzed by the MaxExpect algorithm. Variant miR196a2-T shows an additional loop in the mature miR196a2 stem-loop structure (arrow head) **A.** Quantitative PCR was performed to compare mRNA levels of predicted miR196a2 downstream targets (Supplementary Table S3) in endometrial HEC1A and RL95-2 cells transfected with miR196a2-C or miR196a2-T vectors. Data were represented as means of triplicates with standard variations. **B.** *P<0.05; **P<0.01; ***P<0.001.

Previous studies showed that MiRSNPs alter miRNA secondary structure and stability, resulting in gene expression and cellular signaling network changes, which may subsequently lead to cancer development [34, 35]. To address this, we used the MaxExpect algorithm (http://rna.urmc.rochester.edu/RNAstructureWeb/Servers/MaxExpect/MaxExpect.html) to predict possible structural changes in resulting pre-miRNAs and miRNAs [36]. Although miRNA-100 is upregulated in endometriosis tissues compared with normal or eutopic endometrium [17, 18], a recent study described its tumor-suppressive role in cancer through an untypical EMT process [37]. We therefore focused on the effects of genetic variations at rs11614913 in *MIR196A2* (Figure 2A). Consistent with a previous study using free-energy analysis [38], a C to U(T) change in pre-miR196a2 generated an additional loop in the hairpin structure, leading to reduced stability and a smaller amount of the mature miR196a2 [31]. Quantitative PCR (qPCR) revealed that 6 of the 14 top-ranked target genes (Supplementary Table S3) in HEC1A (with a T/C genotype background) and 9 of the 14 genes in RL95-2 (T/T genotype) were upregulated in endometrial cells transfected with miR196a2-T vector (T allele at rs11614913) as compared to cells transfected with miR196a2-C vector (C allele at rs11614913) (Figure 2B), indicating insufficient silencing by the C to T substitution. Such target gene expression changes, although minimal, might alter downstream signaling.

**Figure 3:**
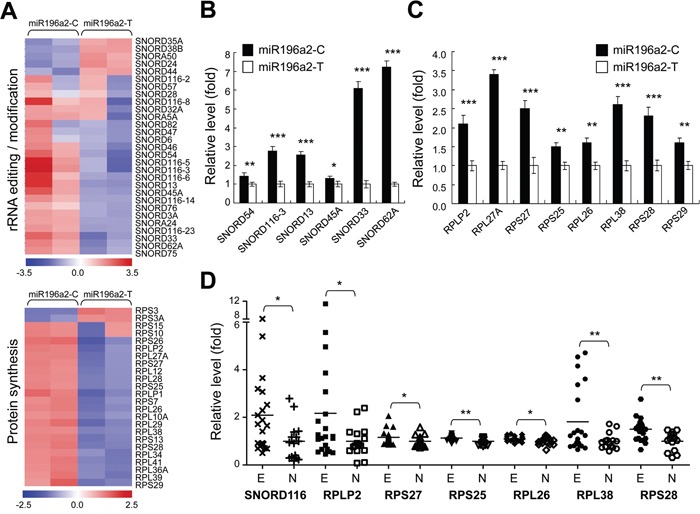
Genetic variations at rs11614913 in MIR196A2 lead to rRNA editing and protein synthesis malfunction in endometrial cells Endometrial HEC1A cells were transfected with miR196a2-C or miR196a2-T vectors. Microarray analysis showed that levels of snoRNAs (upper) and RPs (lower) were affected by rs11614913 variants **A.** Quantitative PCR was performed to validate expression of snoRNA **B.** and RP **C.** genes in transfected cells. Data were represented as means of triplicates with standard variations. Microarray data from the GEO databank (GSE6364) was used to assess expression of selected snoRNA and RP genes in endometriosis lesions (n=21) and normal endometrium (n=16) **D.** *P<0.05; **P<0.01; ***P<0.001.

To study the biological relevance of *MIR196A2* polymorphisms in endometrial cells, cells transfected with either miR196a2-T or miR196a2-C vectors were subjected to gene expression profiling by microarray analysis. The miR196a2-C vector induced >1.5 fold changes in a majority of known C/D snoRNAs (Figure 3A, *upper*). Nearly half the known human RPs were also moderately increased (fold change > 1.3; Figure 3A, *lower*). To confirm the microarray data, we assessed snoRNAs and RPs with fold changes > 2 by qPCR. Most snoRNAs showed expression patterns consistent with the microarray data, although SNORD54 and SNORD45A levels were lower as measured by qPCR (Figure 3B–3C). Among the highly expressed RPs, 60S acidic ribosomal protein P2 (RPLP2), RPL27A, RPS27 (also known as metallopanstimulin-1, MPS-1) and 60S ribosomal protein L38 (RPL38), were validated to be regulated by the miR196a2-C vector.

To define the clinical significance of our findings, microarray data from the GEO databank (accession number: GSE6364) were utilized to analyze expression of the selected snoRNAs and RPs in clinical endometriosis lesions and normal endometrium. Six out of eight RPs were found to be upregulated in endometriosis tissues (Figure 3D). Due to limitations of the probe-set design, small nucleolar RNA C/D box 116 (SNORD116) was the only selected snoRNA gene found in the dataset with higher levels in endometriosis tissues compared to controls. Among the selected genes, SNORD116, RPLP2, RPL38 and 40S ribosomal protein S28 (RPS28) were most elevated ones in endometriosis patients (Figure 3D), which was consistent with our *in vitro* experimental findings using miR196a2-C vector. These results indicate an overall activation of ribosome biogenesis during endometriosis development.

### Ribosome biogenesis upregulation triggers endometriosis progression

**Figure 4:**
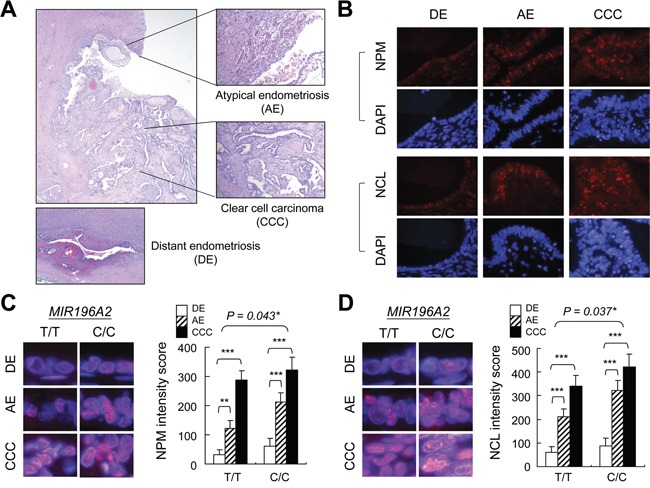
Ribosome biogenesis upregulation during endometriosis progression Tissue sections with contiguous atypical endometriosis and ovarian clear cell carcinoma **A.** were genotyped (5 blocks for C/C and 3 blocks for T/T at rs11614913 in *MIR196A2*) and prepared for anti-NPM and anti-NCL staining **B.** The representative staining images were from tissue blocks with C/C genotype at rs11614913 in *MIR196A2*. Staining was scored as described in Materials and Methods and represented as means of 100 nucleoli with standard variations. Magnified images indicate nucleolus (anti-NPM) **C.** and DFC (anti-NCL) **D.** staining. Distant endometriosis from the same patient was used as the control **A-D.** *P<0.05; **P<0.01; ***P<0.001.

Ribosome biogenesis is the greatest energetic and metabolic expenditure that takes place in the cell nucleolus, especially in cancer cells. Structural-functional studies have revealed that nucleolar abnormalities correlate with cancer development and represent an adaptation to the new metabolic characteristics acquired by transformed cells [39, 40]. Therefore, the activation of ribosome biogenesis might be a driving force triggering malignant transformation during endometriosis progression. To test this hypothesis, we collected five clear cell ovarian carcinomas carrying the risk C/C genotype at rs11614913 in *MIR196A2* and three samples carrying the reference T/T genotype for immunofluorescence staining (Figure 4A). We detected total active nucleoli using anti-nucleophosmin (NPM) antibodies, and the dense fibrillar component (DFC), a region with highly active ribosome biogenesis, using anti-nucleolin (NCL) antibodies. Our results indicated that contiguous atypical endometriosis adjacent to cancer tissues had greater NCL and NPM staining intensities compared to distant endometriosis lesions (Figure 4B–4D). Consistent with this, cancerous tissue nucleoli had greatly enlarged total areas (anti-NPM) with activated ribosome biogenesis (anti-NCL) (Fig 4B–4D). Of note, the tissue blocks carrying the T/T genotype showed a weaker staining intensities than those carrying the C/C genotype. However, the increasing patterns in are similar between these two groups (Fig 4C-4D). Our data provide evidence that increased nucleoli and enlarged DFC morphology indicate an unfavorable transformation from endometriosis to atypical endometriosis and finally ovarian cancer.

### RNA polymerase 1 inhibition suppresses cell growth and mobility of ovarian clear cells

**Figure 5:**
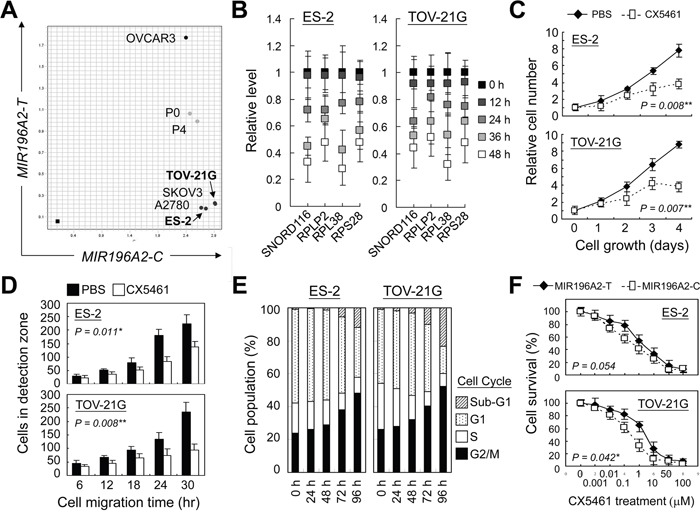
RNA polymerase 1 inhibition suppresses cell growth and mobility of ovarian clear cells Ovarian cancer cell lines were genotyped by *Taqman* method as described in Materials and Methods. Cancer cell lines from ovarian clear cell carcinoma, ES-2 and TOV-21G, were found to possess C/C genotype at rs11614913 in *MIR196A2* **A.** Cells were treated with the RNA polymerase 1 inhibitor, CX5461, at a final concentration of 25 nM. CX5461 effects on miR196a2 downstream effectors were measured by qPCR at the indicated time points. Data were represented as means of triplicates with standard variations **B.** CX5461 effects on cell growth were assessed via MTT assay **C.** Cell mobility was measured using wound-healing migration assays. Cells migrating into the detection zone were counted and averaged from eight replicates **D.** Treated cells were analyzed at the indicated time points by flow cytometry using propidium iodide DNA staining **E.** Drug sensitivity toward CX5461 treatment was measured on day 4 in cells transfected with miR196a2-C or miR196a2-T vectors **F.**

Upregulation of snoRNAs and RPs has been linked to accelerate cell proliferation, thus has been suggested as a therapeutic target for cancer treatment [29, 41]. Two ovarian clear cell carcinoma cell lines with wild type *TP53* genetic status [42], ES-2 and TOV-21G, were used to investigate the efficacy of anti-ribosome biogenesis therapy by treating cells with CX5461, an RNA polymerase I inhibitor. Genotyping assay confirmed the C/C genotype at rs11614913 in *MIR196A2* in both cell lines, indicating these two as good models for this study (Figure 5A). Expression of the selected snoRNAs and RPs reduced following 24 h of CX5461 treatment (Figure 5B). Cell proliferation and mobility rates were decreased with inhibition of DNA synthesis after 48 h of CX5461 treatment (Fig 5C–E). CX5461 treatment triggered cell arrest at G2/M-phase (48.1% in ES-2 cells and 52.3% in TOV-21G cells) and cell death (12.3% in ES-2 cells and 23.4% in TOV-21G cells) after 96 h of CX5461 treatment (Figure 5E). Cells transfected with miR196a2-T vector slightly increased drug tolerance to CX5461 treatment, suggesting differential response activity determined by *MIR196A2* variants (Figure 5F).

## DISCUSSION

Functional MiRSNPs impact human disease, including cancer development [12–14]. We assessed seven cancer-related MiRSNPs and found that genetic variations at rs11614913 in *MIR196A2* are associated with endometriosis development and progression. The rs11614913 C allele correlated with a greater tendency for patients to develop infertility and severe pain. Functional characterization in endometrial cells demonstrated a role for this risk allele in ribosome biogenesis via regulating expression of multiple snoRNAs and RPs. These snoRNAs and RPs were generally upregulated in endometriosis lesions as compared to normal endometrium, suggesting that active ribosome biogenesis in cell nucleoli drives endometriosis. Inmmunofluorescent staining against NPM and NCL further confirmed that changes in nucleolar integrity correlate with aggressive progression from endometriosis to atypical endometriosis and clear cell ovarian cancer. Treatment with CX5461, an RNA polymerase 1 inhibitor, inhibited cell proliferation and migration in ovarian clear cells that possess the risk C/C genotype of rs11614913, and triggered cell cycle arrest at G2/M phase and apoptosis. To our knowledge, this is the first report to address the roles of *MIR196A2* genetic variants in endometriosis development and malignant transformation.

The functional SNP rs11614913 in *MIR196A2* is associated with cancer development, including lung and breast cancers [31–33]. Although some discrepancies exist across different cancer types and ethnic groups, most studies associate the CC or CT genotypes at rs11614913 with poorer patient outcomes, indicating the C allele as the risk allele. Consistent with our finding, rs11614913-C is reportedly more structurally stable than rs11614913-T, and is correlated with increased mature miR196a2 in clinical specimens [31]. *MIR196A2* is located in the *HOXC* cluster region on chromosome 12. Nearly one-third of known or putative miR196a2 targets (Supplementary Table S3) are members of the Hox gene family, which encodes homeodomain-containing transcription factors essential for embryonic development [43, 44]. Hox proteins participate in cell division, adhesion/migration and apoptosis [45, 46], and dysregulation of these proteins has been linked to endometriosis development, embryo implantation and malignancy [47–50]. However, most Hox proteins are sensitive to steroid hormones, including the clinically used hormone drugs, and their levels change along with the menstrual cycle [47, 48]. This might explain why we did not see consistent Hox expression patterns in clinical specimens.

On the other hand, an overall increase in snoRNAs and RPs as the downstream effectors of miR196a2 suggests enhanced ribosome activity crucial for cell proliferation and the expansion of endometriosis tissue. Our data indicated that rs11614913-C enhanced expression of SNORD116, RPLP2, RPS27, RPS25, RPL26, RPL38 and RPS28, all of which were upregulated in clinical specimens. Florescent staining against both NPM (active nucleoli) and NCL (DFC regions in the nucleoli) revealed that ribosome biogenesis was more active in contiguous atypical endometriosis than in distant endometriosis, and greater staining patterns can be found in cancerous tissues. Thus, our study suggests the point that active ribosome biogenesis could be a driving force for malignant transformation during endometriosis development and progression. However, limited tissue blocks were available for this study, which may not provide a comprehensive scenario of how upregulation of ribosome biogenesis promotes endometriosis progression.

Previous studies support our finding that overexpression of RPs contributes to cell transformation and could be utilized as prognostic markers for human cancers [51–55]. Ribosomal P protein (RPLP0, RPLP1, RPLP2) expression was previously shown to correlate with invasiveness and metastasis in gynecologic tumors [51]. Although limited information is available regarding the functions of SNORD116, a C/D box snoRNA that controls the 2′-*O*-ribose methylation of rRNAs, accumulating evidence implicates snoRNAs in the control of cell fate and carcinogenesis through a bypassing of ribosomal/oncogenic stress responses [25, 56, 57]. For example, upregulation of C/D box snoRNAs was reported as a common feature in breast and prostate cancers [57].

In addition to their key functions in ribosome assembly and protein synthesis, snoRNAs and RPs play novel roles outside cell nucleoli, regulating the activity and function of other oncogenes or tumor suppressors. Several downstream effectors of rs11614913-C, including RPS27, RPL26, RPS25 and RPL26, participate in the MDM2-p53 feedback loop upon ribosomal/oncogenic stress [27]. Disruption of rRNA synthesis and editing/processing, such as by chemical inhibition of RNA polymerase I, triggers MDM2 degradation and stabilizes/activates p53, leading to cell apoptosis or senescence [27, 56, 58]. Similarly, specific siRNAs against C/D box snoRNAs suppressed cell cycle progress and reduced tumor growth by activating p53 [57]. With emerging roles for RNA editing in cancer development [59], targeting rDNA transcription and the nucleolus is a feasible cancer treatment strategy, and has shown efficacy against hematological malignancies [29, 41].

Notably, human cancers exhibit differential sensitivity to anti-RNA polymerase 1 therapy, depending largely on *TP53* status [41]. Genetic analysis has shown that *TP53* mutations rarely occur (~10%) in endometriosis-associated ovarian cancers, and are considered as late genetic events during endometriosis progression if they occur [60, 61]. Our study indicates enhanced ribosome biogenesis activity during endometriosis development, and this activity is more pronounced during the malignant transition. This suggests that anti-RNA polymerase I therapy may be efficacious for treating endometriosis and associated ovarian cancers. Additionally, genetic variations at rs11614913 in *MIR196A2*, along with upregulation of snoRNAs and RPs associated with ribosomal biogenesis, may be useful prognostic indicators in endometriosis patients.

It is still unknown how genetic variation in a miRNA can promote upregulation of genes involved in ribosome biogenesis, especially C allele at rs11614913 can form more stable and abundant mature miR196a2. In addition, the selected snoRNAs and RPs in this study are not putative direct targets of miR196a2 based on the degree of complementarity between the target site and the miRNA. Interestingly, recent studies did provide evidence that miRNAs can promote specific gene upregulation through direct or indirect mechanisms [62]. These clues support the possible involvement of other factors in miR196a2-mediated upregulation of ribosome biogenesis which is worth a further investigation.

## MATERIALS AND METHODS

### Study population

The study population consisted of 218 individuals who were pathologically diagnosed with endometriosis and underwent laparotomy or laparoscopy at the China Medical University Hospital (CMUH) in Taiwan. Patient disease-related fertility statuses were verified by clinical reports. Endometriosis stages were classified using the guidelines of the American Society of Reproductive Medicine (ASRM): stage 1, minimal; stage 2, mild; stage 3, moderate; stage 4, severe [63]. The control group consisted of 202 healthy women age-matched to the patient group, and received regular physiological examinations at the same hospital. People who showed ovarian cysts detected by ultrasound or any one of the endometriosis-associated symptoms, even though the results of their health checkups were normal, were excluded from this study. This study was approved by the Institutional Review Board (IRB) at the CMUH, with informed consent from each participant.

### Genotyping of single nucleotide polymorphisms

Genomic DNA was extracted from peripheral blood leukocytes or cell pellets according to standard protocols (Genomic DNA kit; Qiagen, Valencia, CA, USA). DNA fragments containing the SNP sites were amplified by PCR using the *Taqman* SNP genotyping assay system (Applied Biosystems Inc. Carlsbad, CA, USA) as previously described [64]. Probe IDs for the seven selected SNPs are listed in Supplementary Table S1. A perfect match between the probe and the tested DNA fragment generated a positive signal. Genetic variations were detected via the fluorescence signals of PCR products.

### Statistical analysis

Allelic and genotypic frequency distributions for the seven SNPs in patients and controls were determined by chi-square analysis using SPSS software (version 10.0, SPSS Inc. Chicago, IL, USA) and expressed as percentages of the total number of alleles and genotypes. Odds ratios (ORs) were calculated for allelic and genotypic frequencies with 95% confidence intervals (95% CIs), using the most frequent allele as the reference. Combined risk analysis and differences between different drug/vector-treated cells were assessed via one-way ANOVA. Simple t-test was used to evaluate whether two groups with different treatments are equal or not.

### Cell culture, gene transfection, cell sorting and functional study

Endometrial cells (HEC1A and RL95-2) and ovarian clear cells (ES-2 and TOV-21G) were purchased from Bioresource Collection and Research center (BRCR), Taiwan. Vector pCMV-MIR (Origene, Rockville, MD) containing a green fluorescein protein (GFP) reporter gene was used to construct the miR196a2-C plasmid. A mutation (C to T) was introduced into the miR196a2-C plasmid at rs11614913 to generate the miR196a2-T using the QuikChange II site-directed mutagenesis kit (Agilent Technologies Inc., Santa Clara, CA). Sequences of resulting vectors were verified by direct sequencing ((Supplementary Figure S1). For microarray analysis, 5×10^5^ HEC1A endometrial cells were seeded in 6-cm dishes. Plasmids were introduced into cells using Lipofectamine (Invitrogen, Waltham, MA) as per the manufacturer's protocol. G418 at a final concentration of 200 μ g/ml was added to culture medium 24 h post-transfection to select for positively transfected cells. At 48 h post-transfection, positive cells were sorted by GFP level via flow cytometry (Becton Dickinson, San Jose, CA), such that over 90% of cells were positively transfected. Sorting efficiencies were checked by counting fluorescent cells under a microscope. For anti-ribosome biogenesis assays, including cell growth, cell migration and cell cycle analysis, ovarian clear cells were maintained for five days in culture medium with or without RNA polymerase I inhibitor, CX5461 (Selleckchem, Houston, TX).

### Microarray experiment

Total RNA was prepared from sorted cells with TRIzol Reagent (Invitrogen) following the manufacturer's protocol. RNA quality was assessed using the Agilent Bioanalyzer (Agilent Technologies). Total RNA from each sample was processed for reverse transcription and fragmentation, followed by hybridization onto a GeneChip® human gene 1.0 ST Array (Affymetrix Inc, Santa Clara, CA). Gene chips were scanned after the wash step, and raw gene expression data in the generated CEL files were normalized and processed using the dChip algorithm [65]. Further clustering and visualization were performed using the TM4 algorithm [66]. Quantitative PCR analysis was performed to validate microarray data using the same RNA samples. To study clinical relevance, the microarray dataset (accession number: GSE6364) that comprises gene expression profiles of 16 normal endometriums and 21 endometriosis lesions was downloaded from the GEO databank (http://www.ncbi.nlm.nih.gov/gds). Data mining was performed by normalizing expression levels of a selected gene in normal endometriums as 1.0.

### Immunofluorescence staining

Eight paraffin blocks showing continuous histopathological transition from distant endometriosis, contiguous atypical endometriosis and ovarian clear cell carcinomas were selected for sectioning. Five of the blocks were genotyped as carrying the C/C genotype, three as carrying T/T genotype at rs11614913 in *MIR196A2*, and were utilized for this study. Immunofluorescence staining was performed to detect active cell nucleoli and ribosome biogenesis activity using 1:100 rabbit anti-nucleophosmin (anti-NPM; ab52644) and anti-nucleolin (anti-NCL; ab129200) monoclonal antibodies (Abcam PLC, Cambridge, MA). Immunostaining was independently scored by two pathologists, and specific nucleolus staining was scored as: negative (0), weakly positive (1+), moderately positive (2+) or strongly positive (3+). We used a combination of the percentage of positively stained cells and the intensity of nucleolus staining for statistical analysis. The H-score = ΣPi xi was calculated, where i is the intensity of the stained tumor cells (0 to 3+) and Pi is the percentage of the stained tumor cells for each intensity group (0 to 100%) as previously described [67]. For discordant cases, a third investigator was brought in to score and the final intensity score was determined by the majority scores.

## SUPPLEMENTARY MATERIALS FIGURE AND TABLES


