# Supplementary material for: Up-regulation of ribosome biogenesis by MIR196A2 genetic variation promotes endometriosis development and progression
Source: Oncotarget. 2016 Sep 15;7(47):76713–25. doi: 10.18632/oncotarget.11536 (PMC5363543; doi:10.18632/oncotarget.11536)
Supplement: Supplementary file 1 [file oncotarget-07-76713-s001.pdf]

Up-regulation of ribosome biogenesis by *MIR196A2* genetic variation promotes endometriosis development and progression

SUPPLEMENTARY FIGURE AND TABLES

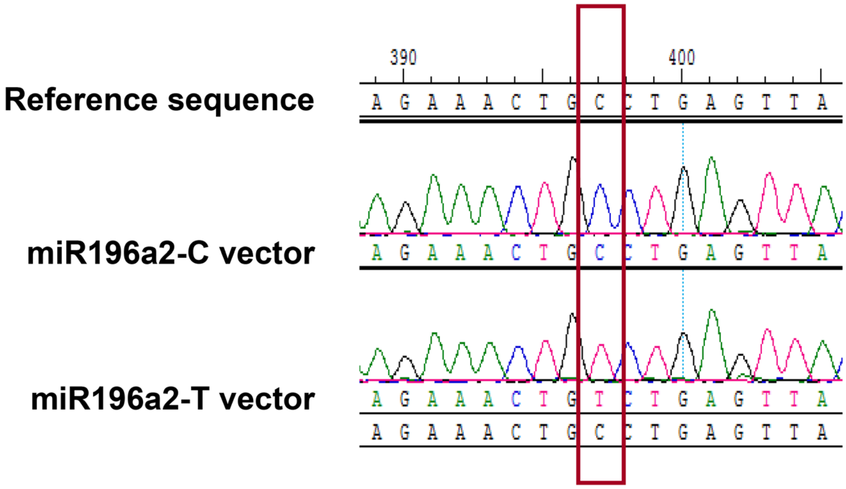

Supplementary Figure S1: Sequence alignment of miR196a2 expression vectors. The miR196a2-C sequence was cloned into the pCMV-MIR vector which contains a green fluorescein protein reporter gene. A mutation (C to T) was introduced into the miR196a2 plasmid at rs11614913 to generate the miR196a2-T vector using site-directed mutagenesis. The sequences of the resulting vectors were verified by direct sequencing.

Supplementary Table S1: Summary of the seven cancer-related MiRSNPs

SNP	Location in miRNA	Associated cancer type	Reference (PMID)	Allele frequencies[a] (%)	ABI probe ID
rs1834306	Pri-miR-100	Colon	20585341	A: 43 G: 57	C__11483095_10_F
rs2910164	Pre-miR-146a	Gastric<br>Lung<br>Breast [a]/ovarian	22455393<br>22818121<br>18660546	G: 43 C: 57	C__15946974_10
rs11614913	Pre-miR-196a2	Breast[a]<br>Lung<br>liver	19567675<br>19293314<br>21692953	C: 45 T: 55	C__31185852_10
rs7372209	Pri-miR-26a1	Colon	20585341	T: 32 C: 68	C__29123986_10
rs895819	Pre-miR-27a	Gastric<br>Breast	20666778<br>19921425	C: 29 T:71	C__11483095_10_F
rs6505162	Pre-miR-423	Breast	22593246	A: 16 C: 84	C__11613678_10
rs3746444	Pre-miR-499	Head and neck<br>Breast	20549817<br>18634034	C: 17 T: 83	AHQIU1L [b]

[a]Allelic type frequencies provided by HapMap database (www.hapmap.org) for Han Chinese in Beijing (CHB).
[b]A customer-designed probe.

Supplementary Table S2: Combined risk analysis of endometriosis and the endometriosis-related infertility using MiRSNP markers

Association	Gene (risk genotype or allele)	Risk score	No.(%) of presence	No. (%) in absence	*P*-value	OR(95% CI)
Endometriosis	miR-196a2 (CC or CT)	2	26 (13.5)	10 (5.0)	4.1×10^{-13}	8.84(4.06-19.2)
	miR-100 (AA)	1	136 (70.8)	87 (43.7)		5.31(3.26-8.66)
		0	30 (15.6)	102 (51.3)		1.00
Infertility	miR-196a2 (C)	2	18 (36.0)	36 (12.6)	1.4×10^{-3}	N/A
	miR-100 (A)	1	32 (64.0)	184 (64.3)		N/A
		0	0 (0.0)	66 (23.1)		1.00

Supplementary Table S3: The predicted downstream targets regulated by miR196a2[a]

Target gene	RefSeq.	Gene description	Target rank		
			miRDB[b]	TargetScan[c]	microRNA[d]
AQP4	NM_001650	aquaporin 4	3	17	NA
CCDC47	NM_020198	coiled-coil domain containing protein 47	12	15	NA
CCNJ	NM_001134375	cyclin J	22	32	14
GATA6	NM_005257	transcription factor GATA-6	6	20	NA
HOXA5	NM_019102	homeobox protein Hox-A5	19	23	NA
HOXA7	NM_006896	homeobox protein Hox-A7	17	2	1
HOXA9	NM_152739	homeobox protein Hox-A9	NA	4	5
HOXB7	NM_004502	homeobox protein Hox-B7	5	16	NA
HOXC8	NM_022658	homeobox protein Hox-C8	10	1	2
MAP3K1	NM_005921	mitogen-activated protein kinase kinase kinase 1	13	11	15
NR2C2	NM_003298	nuclear receptor subfamily 2 group C member 2	4	12	NA
SLC9A6	NM_001042537	sodium/hydrogen exchanger 6	2	3	4
SMC3	NM_005445	structural maintenance of chromosomes protein 3	28	NA	3
ZMYND11	NM_006624	zinc finger MYND domain-containing protein 11	1	8	9

[a]The downstream targets of miR196a2 were predicted by overlapping the prediction results from three different algorithms. The genes that were ranked within the top-50 list by any two algorithms were considered as the potent target genes.
[b]miRDB (http://mirdb.org/miRDB).
[c]TargetScan (http://www.targetscan.org).
[d]microRNA (http://www.microrna.org/microrna/home.do).
